# The Influence of Fungicide Treatments on Mycobiota of Grapes and Its Evolution During Fermentation Evaluated by Metagenomic and Culture-Dependent Methods

**DOI:** 10.3390/microorganisms7050114

**Published:** 2019-04-26

**Authors:** Alice Agarbati, Laura Canonico, Leonardo Mancabelli, Christian Milani, Marco Ventura, Maurizio Ciani, Francesca Comitini

**Affiliations:** 1 Department of Life and Environmental Sciences, Polytechnic University of Marche, Via Brecce Bianche, 60121 Ancona, Italy; alice.aga@libero.it (A.A.); l.canonico@univpm.it (L.C.); 2 Laboratory of Probiogenomics, Department of Genetics, Biology of Microorganisms, Anthropology and Evolution, University of Parma, 43100 Parma, Italy; leonardo.mancabelli@studenti.unipr.it (L.M.); christian.milani@unipr.it (C.M.); marco.ventura@unipr.it (M.V.)

**Keywords:** mycobiota of grapes, organic treatment, conventional treatment, Next Generation Sequencing, culture-dependent method

## Abstract

The present study evaluated the impact of organic and conventional fungicide treatments compared with untreated samples (no fungicides were used) on the grape berry yeast community of the Montepulciano variety. The yeast dynamics during the spontaneous fermentation using culture-dependent and -independent methods was also evaluated. Results showed a reduction of yeast biodiversity by conventional treatments determining a negative influence on fermenting yeasts in favor of oxidative yeasts such as *Aerobasidium pullulans*. *Starmerella bacillaris* was significantly more present in organic samples (detected by next generation sequencing (NGS)), while *Hanseniaspopa uvarum* was significantly less present in untreated samples (detected by the culture-dependent method). The fermenting yeasts, developed during the spontaneous fermentation, were differently present depending on the fungicide treatments used. Culture-dependent and -independent methods exhibited the same most abundant yeast species during the spontaneous fermentation but a different relative abundance. Differently, the NGS method was able to detect a greater biodiversity (lower abundant species) in comparison with the culture-dependent method. In this regard, the methodologies used gave a different picture of yeast dynamics during the fermentation process. The results indicated that the fungal treatments can influence the yeast community of grapes leading must fermentation and the final composition of wine.

## 1. Introduction

The fruit surface, and specifically grape berries, is a complex and specific ecologic niche colonized by different microorganisms such as filamentous fungi, yeasts and bacteria with different physiological characteristics [1,2,3]. Several environmental factors such as geographical region, climatic condition (temperature, humidity, UV radiation, etc.), availability of nutrients and farming treatments could influence the composition of microbiota [4,5,6,7,8,9]. The possible interactions among the factors could also affect biodiversity and stability of microbiota, grapevine health and as final consequence, the quality of wines [1,10]. Furthermore, bees and wasps can play an important role on the occurrence of microorganisms, influencing the transfer from one side to the other, including grape surfaces [11,12]. The fungi species often found on grapes are saprophytic molds such as *Cladosporium* spp., *Penicillium* spp., *Aspergillus* spp., that do not have the ability to grow in wine and have no direct influence on winemaking. Instead, there are other microorganisms, such as yeasts, acetic acid bacteria and lactic acid bacteria that are part of the so-called wine microbial consortium (WMC) because they are able to survive or grow in grape juice and wine [1] and could influence its final quality. Concerning yeasts, they could be grouped in: species easily controllable or technologically irrelevant, oxidative or weakly fermenting species present at pre-fermentation stages and/or at the beginning of fermentation (such as *Hanseniaspora* spp., *Candida* spp., *Pichia* spp., *Metschnikowia* spp.), strong fermenting yeasts liable for wine fermentation (belonging to *Saccharomyces* spp.) [1,13], and spoilage yeasts (such as *Dekkera bruxellensis, Zygosaccharomyces bailii*) responsible for wine alterations [14,15]. Several studies reported that one of the most important factors that influences microbial community composition associated with grape berries are the vineyard agronomic practices [5,16,17,18,19,20,21,22,23]. Indeed, Cordero-Bueso and co-workers [20] showed a greater biodiversity of yeast species when the vineyard was treated with organic practices instead conventional ones. On the contrary, Comitini and Ciani [5] found a reduction in the yeast diversity when organic fungicides were applied. More recently, Escribano-Viana et al. [23] found that the bio-fungicide did not show significant impact on the wine microbiota whereas the chemical fungicide caused a reduction of microbial community richness and diversity.

Regarding *S. cerevisiae* presence, Ganga and Martinez [18] showed no effect on the enumeration of this fermenting yeast after fungicide application. Tello et al. [21] described beneficial effect of organic farming system in terms of diversity and abundance as well as on *S. cerevisiae* strains’ biodiversity. Milanović and co-workers [22] found greater *S. cerevisiae* strain biodiversity in conventional samples than organic ones.

To investigate the microbial composition of grape berries and to monitor their evolution during must fermentation, it is relevant to understand the relationship among the different microorganisms that cohabit during the winemaking process [7,24]. The use of culture-dependent techniques allow the detection of the more abundant culturable microorganisms associated with grape berries and wine. However, there are many viable, but non-culturable wine microorganisms, that could not be analyzed using conventional techniques. Therefore, these techniques lead to incomplete knowledge about the composition and dynamics of the microbial community involved in winemaking [24,25,26]. Recent advances in sequencing technologies based on culture-independent techniques allow to capture a large proportion of microbes using high-throughput next generation sequencing obtaining a more complete microbial ecology picture, even if the methodology and the interpretation of data should be set up [2,27,28,29].

In this study, we investigated the yeast community of the grape berry surface of the Montepulciano variety, subjected to different fungicide treatments using both culture-dependent and -independent approaches. The yeasts’ dynamic composition during the spontaneous fermentation carried out in sterile conditions was also evaluated.

## 2. Materials and Methods

### 2.1. Vineyard Treatments and Grape Sampling

The grapes used in this study were obtained from the Montepulciano vine, an autochthonous vineyard of the center of Italy. In particular, these vines are situated in Sirolo locality (43°31′20″ N, 13°36′53″ E; 97 m altitude), in the Marche region and during the sampling time (October 2016) the main climatic conditions were 14.9 °C for air temperature, 82% humidity and there were 15 rainy days. The vineyard included three blocks of rows and each block has employed different agronomic practices like as organic, conventional and with no treatment. The distance between the blocks was about 1 km to exclude cross-contaminations between the treatments.

The organic treatment was performed in 15 consecutive applications from April 20 to August 17 and included a Bordeaux mixture (20 g L^−1^ of copper (II) sulfate + 13 g L^−1^ of calcium hydroxide with pH 6.6) and sulfur (Microthiol disperss, UPL EUROPE Ltd., Warrington WA3 6YN, Great Britain).

The conventional treatment was performed in nine consecutive applications from March 10 to July 17 and included chemical compounds such as spiroxamina (Prospher300 CS, Bayer Crop Science, Monheim am Rein, Germany), copper-oxychloride (Coprantol, Sygenta Italia Spa, Casalmorano, Cremona, Italy), sulfur (Tiovit jet, Sygenta Italia Spa, Casalmorano, Cremona, Italy), fosetyl-Al+copper sulfate (R6 Erresei Bordeaux WG, Bayer Crop Science, Monheim am Rein, Germany), Metalaxyl-M14+ copper-oxychloride (RidomilGold, Sygenta Italia Spa, Casalmorano, Cremona, Italy), quinoxyfen + myclobutanil + coformulants (Arius System Plus, Dow AgroSciences, Indianapolis, Indiana, USA), copper sulfate and sulfur.

The grape samplings were collected in sterile plastic bags of about 1 kg of undamaged ripe grape bunches for each sample and were immediately transported to the laboratory on ice for processing. In particular, seven organic (MO), seven conventional (MC) and three not treated (MNT) samples were collected.

### 2.2. Grape Juice Spontaneous Fermentations

The grapes, as soon as they arrived in the laboratory, were crushed and shaken at 120 rpm for 30 min on a MAXQ 4450 shaker (Thermo Fisher Scientific, Waltham, Massachusetts, USA) under sterile conditions. Part of each grape juice was used for yeast counts and total microbial DNA extraction while the remaining fresh must (skin of grape included) was used for the set up of spontaneous fermentation. The spontaneous fermentations were carried out in 250 mL sterile Erlenmeyer flasks closed with Pasteur bungs to allow CO_2_ to escape and placed at 25 °C under static conditions. Monitoring of the microbial population composition at the beginning and their evolution at the 7th and 15th day from the start to the fermentation was done through viable counts and high-throughput next generation sequencing (NGS).

### 2.3. Viable Counts, Yeast Isolations and Analytical Procedures

The total yeast enumeration was carried out by taking 1 mL of fresh musts and samples at the 7th and 15th day of fermentation, serial decimal dilutions in sterile water were prepared and spread on Wallerstein (WL) nutrient agar (Merck KGaA, Germany) supplemented with 0.02% biphenyl (Sigma-Aldrich, Saint Louis, Missouri, USA) and 0.005% chloramphenicol (Thermo Fisher GmbH, Germany) to prevent mold and bacteria growth respectively. The plates were incubated at 25 °C for five days and those that contained between 30 and 300 colonies were analyzed for cell counts, macro- and micro-morphological characteristics and used for yeast isolation. The yeast isolation was carried out on YPD agar (1% Yeast Extract, 2% Peptone, 2% d-glucose, and 2% Agar) collecting approximately 10% of the colonies per plate [22,30]. These yeasts were maintained in 40% (v/v) glycerol at −80 °C. To determine the sugar concentrations, a specific enzyme kit (Megazyme International Ireland, Wicklow, Ireland) was used. Ethanol content and volatile acidity were measured using the current analytical methods according to the Official European Union Methods [31].

### 2.4. Yeasts Identification

The 700 isolated strains were grouped based on the same macro- and micro-morphological features and representative isolates were used for genomic DNA analysis according to the method described by Stringini et al. [30]. The internal transcribed spacer ITS1-5.8S rRNA-ITS2 region was amplified by PCR using the primer set ITS1 (5′-TCCGTAGGTGAACCTCGCG-3′) and ITS4 (5′-TCCTCCGCTTTATTGATATGC-3′) [32] as described by Esteve-Zarzoso and co-workers [33]. Horizontal electrophoresis (Bio-Rad, Hercules, USA) was used to analyze the PCR products using 1.5% (*w*/*v*) agarose gel with ethidium bromide, in 0.5x TBE buffer. The representative yeast species were identified by sequencing and through use of the BLAST program [34], the sequences provided were compared with those already present in the data library GenBank (http://www.ncbi.nlm.nih.gov/BLAST). The inclusion of obtained sequences into the NCBI GenBank data library was completed under the accession numbers from MK352017–MK352031 and from MK352058–MK352096.

### 2.5. Total DNA Extraction and Next Generation Sequencing (NGS) Analysis

To obtain the total microbial DNA that represent well mixed microbial consortia of samples, 1 mL of each fresh juice and each sample at the 7th and 15th day of spontaneous fermentation was taken. The total DNA extraction was carried out following the protocol of the Soil Kit DNA Extraction (Qiagen, Hilde, Germany) and the extracts were stored at –20 °C until further analysis.

The presence of fungal genome was confirmed using primer set NL1 (5′-GCATATCAATAAGCGGAGGAAAAG-3′) and NL4 (5′-GGTCCGTGTTTCAAGACGG-3′) to amplify the region 26S rDNA D1/D2 as described by Kurtzman and Robnett [35].

Next Generation Sequencing (NGS) analyses were performed using primers BITS (5′-GAGATCCRTTGYTRAAAGTT-3′) and B58S3 (5′-ACCTGCGGARGGATCA-3′) [36] to amplify the fungal internal transcribed spacer (ITS) region. Library preparation of the samples was carried out using Illumina paired-end kit, cluster generation, and 350-bp paired-end sequencing on an Illumina Miseq (Illumina Inc., San Diego, CA, USA).

### 2.6. NGS Data Processing

The fastq files obtained from sequencing were processed using a custom script based on the QIIME software suite [37]. In detail, paired-end reads pairs were assembled to reconstruct the complete BITS/B58S3 amplicons. Forward reads of unmerged pairs were also included in the analysis. Quality control were retained sequences with a mean sequence quality score >15, while sequences with mismatched primers were omitted. In order to calculate fungal taxonomy, ITS rRNA Operational Taxonomic Units (OTUs) were defined at ≥99% sequence homology using uclust [38]. and OTUs with less than 10 sequences were filtered. All reads were classified to the lowest possible taxonomic rank using QIIME [37] and a reference dataset from the UNITE database [39].

The microbial richness of the samples (alpha-diversity) were calculated with Shannon indexes calculated for 10 sub-samplings of sequenced read pools and represented by rarefaction curves. The alpha-diversity could also be represented by a box-and-whisker plot. In detail, the bottom and top of the box were the first and the third quartiles, and the band inside the box was the median. Moreover, the ends of the whiskers represented the minimum and maximum of all the data of the sample. Similarities between samples (beta-diversity) were calculated by weighted uniFrac [40]. The range of similarities was calculated between the values 0 and 1. PCoA (principal component analysis) presentations of beta-diversity were performed using QIIME [37]. In the PCoA, each dot represented a sample that is distributed in tridimensional space according to its own bacterial composition.

### 2.7. Statistical Analysis

Comparisons between different groups were tested by ANOVA (Analysis of Variance) calculated through SPSS software (www.ibm.com/software/it/analytics/spss/). Moreover, we also calculated the post hoc analysis LSD (least significant difference) for multiple comparisons.

## 3. Results

### 3.1. Effects of Fungicide Treatments on Fungal Community at Harvest Time

#### 3.1.1. Culture-Independent Analysis (NGS)

The fungal population associated with the grape surface of the Montepulciano variety was evaluated by the culture-independent method using Next Generation Sequencing (NGS). Samples MNT, MO and MC were compared.

**Figure 1:**
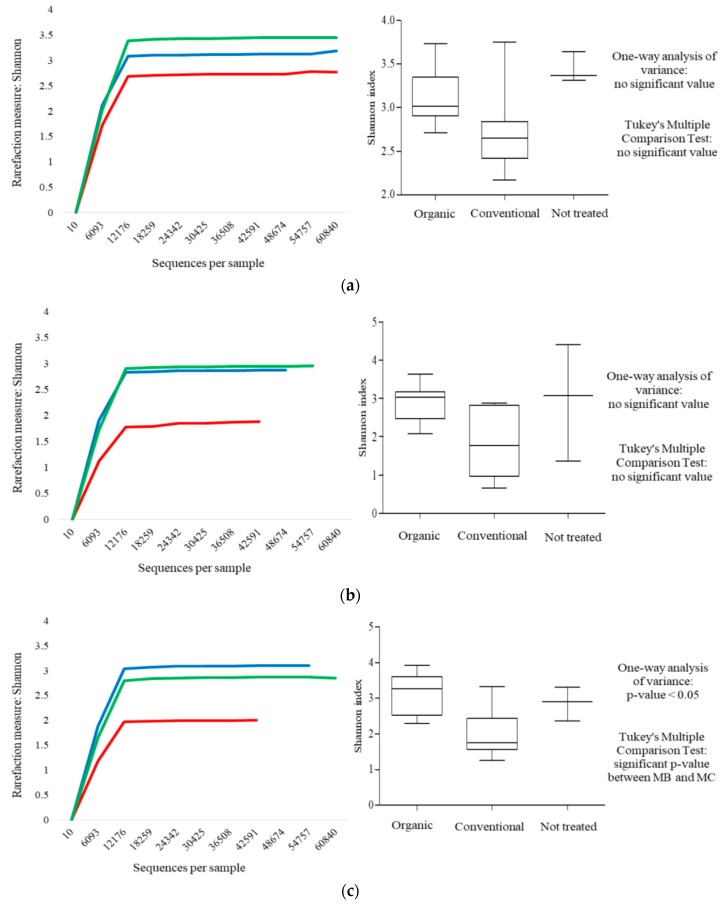
Rarefaction curves (on the left) and box-and-whisker plots (on the right) generated for mean values of fungal internal transcribed spacer (ITS) sequences obtained from MO (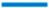), MC (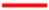) and MNT (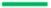) grapes. The results were obtained using the Shannon index. (**a**–**c**) represent rarefaction curves and box-and-whisker plots referred to harvest time and at the 7th and 15th day of spontaneous fermentation, respectively.

Rarefaction curves of fungal population characterizing MO, MC and MNT samples were calculated through the Shannon index, as showed in Figure 1. In all three sample times (harvest, 7th and 15th day of spontaneous fermentation) the plateauing of the three curves related to the diversity indices indicated that the main part of the fungal diversity was detected. In detail, the MNT grapes displayed the highest biodiversity at the harvest time followed by MO and MC ones (Figure 1a). At 7th day of spontaneous fermentation, the biodiversity of MO and MNT grapes was similar, and it was higher than conventional grapes (Figure 1b). At 15th day of fermentation, the MO samples showed the highest biodiversity followed by MNT and MC samples (Figure 1c). Significant differences were found only at the 15th day (between MO and MC) for the higher homogeneity of the samples in comparison with the others.

**Figure 2:**
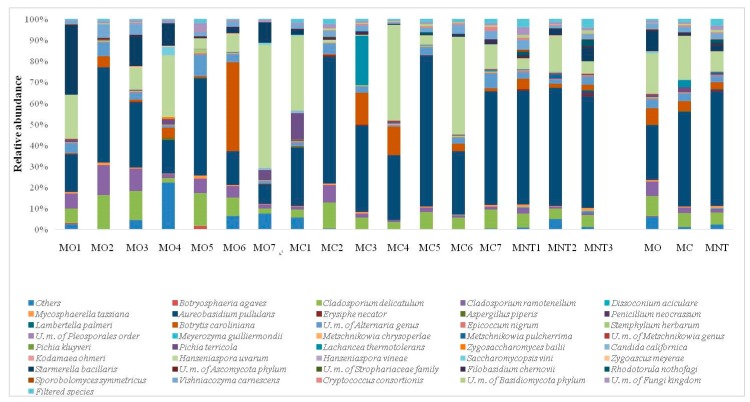
Relative abundance of grape fungal community detected by next generation sequencing (NGS) at harvest time in organic (MO), conventional (MC) and not treated (MNT) samples. The number associated to the samples represent the replicates for each treatment. In the microorganisms legend, *U.m.* abbreviation means undefined microorganism. Only the taxa >0.5% are shown and the taxa ≤0.5% are grouped under the heading Filtered species of the legend. To the right of the graph, mean values of each treatment are represented (MO, MC and MNT).

At harvest time the high-throughput sequencing technology allowed to clearly identify 164 species (yeasts and filamentous fungi). Other fungi were classified only at higher taxonomical level. Unknown fungi were also detected (Figure 2). Mean values of relative abundance revealed that the population was mainly represented by the oxidative yeast-like *Aerobasidium pullulans* followed by the fermentative *Hanseniaspora uvarum* species. The relative abundance of the two species was similar in the MO samples (26.09% of *A. pullulans*, 19.10% of *H. uvarum*), while in MC samples *A. pullulans* (45.12%) predominated over *H. uvarum* (20.81%). *A. pullulans* represented more than 50% of the total fungal population of MNT grapes, while only 9.30% of *H. uvarum* was detected. Conventional treatments affected the presence of *A. pullulans* since significant enhancement of the relative abundance was found in MC samples while *H. uvarum* did not seem influenced by treatments (Table S1). *Starmerella bacillaris* fermentative yeast was positively influenced by organic treatments (9.96%, 0.53% and 2.99% in MO, MC and MNT respectively), while *Lachancea thermotolerans* was found only in MC samples (3.35%). *Zygoascus meyerae* was found in MO and MNT samples (0.23% and 0.17% respectively) and it was not detected in MC samples. *Rhodotorula nothofagi* and *Metschnikowia pulcherrima* were found in MNT samples (1.64% and 0.87%, respectively), while *Pichia terricola* was detected in MO (1.34%) and MC (1.89%) samples. Filamentous fungi such as *Botrytis caroliniana*, *Alternaria* genus, *Cladosporium ramotenellum* and *Cladosporium delicatulum* showed a relevant presence in all samples. Analyzing the mean values of relative abundance, these species exhibited the same trend: they appeared more abundant in MO samples, followed by MC and MNT ones (*B. caroliniana*: 7.87%, 4.89% and 3.10%; *Alternaria* genus: 4.41%, 3.38% and 2.86%; *C. ramotenellum*: 6.93%, 2.46% and 1.55%; *C. delicatulum*: 9.43%, 6.78% and 5.78% in MO, MC and MNT samples respectively). Only *C. ramotenellum* showed a significant increase in relative abundance in MO samples (Table S1). The relative abundances of filamentous fungi found suggest, and confirm, healthy harvested grapes and their low propensity for postharvest spoilage grapes.

#### 3.1.2. Culture-Dependent Analysis

**Figure 3:**
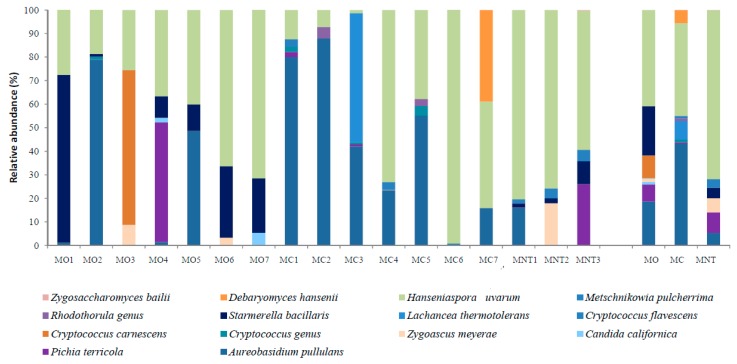
Relative abundance of the grape yeast community detected by the culture-dependent method at harvest time in organic (MO), conventional (MC) and not treated (MNT) samples. The number associated to the samples represent the replicates for each treatment. To the right of the graph, mean values of each treatment are represented (MO, MC and MNT).

The results of the culture-dependent method are shown in Figure 3. The culture-dependent method allowed to detect only 12 yeast species and another two identified at genus level. As showed by NGS analysis, *A. pullulans* and *H. uvarum* were confirmed to be the yeasts mainly detected also in the culture-dependent approach. Likewise, to NGS analysis, MC samples showed higher relative abundance of *A. pullulans* than that showed by MB and MNT samples but not statistically significant (Figure 3 and Table S2). *H. uvarum* was the more abundant species isolated from all samples without significant differences among the MO, MC and MNT samples. As NGS analysis, *S. bacillaris* was mainly found in MO samples (20.94%) and *L. thermotolerans* was found only in MC samples (7.84%). Differently to NGS, *P. terricola* was found in MO, MC and MNT samples. *Z. meyerae* was not detected in MC samples (0.01% of relative abundance with NGS) while it was found in MO and MNT as showed by NGS analysis. By the culture-dependent method, *Rhodotorula* genus was found only in MC samples (1.13%) while *M. pulcherrima* characterized MNT samples showing the same trend described by NGS.

### 3.2. Effects of Fungicide Treatments on Fungal Community at 7th Day of Spontaneous Fermentation

#### 3.2.1. Culture-Independent Analysis (NGS)

**Figure 4:**
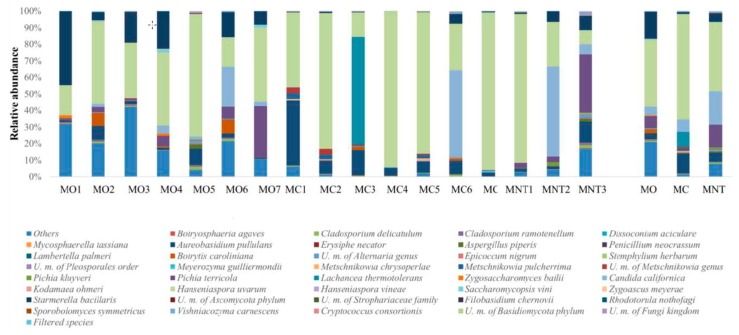
Relative abundance of fungal community detected by NGS at the 7th day of spontaneous fermentation in organic (MO), conventional (MC) and not treated (MNT) samples. The number associated to the samples represent the replicates for each treatment. In the microorganism legend, *U.m.* abbreviation means undefined microorganism. Only the taxa >0.5% are shown, and the taxa ≤0.5% are grouped under heading Filtered species of the legend. To the right of the graph, mean values of each treatment are represented (MO, MC and MNT).

The population dynamic at the 7th day of spontaneous fermentation evaluated by NGS revealed 71 fungal species (Figure 4). Other fungi were classified at higher taxonomical levels. At this stage of fermentation, as expected, *H. uvarum* represented the most abundant specie in MO, MC and MNT samples (40.30%, 63.61% and 41.71% respectively) while the oxidative yeast-like *A. pullulans* decreased in all samples. The same trend was observed for molds, which were found <1% of relative abundance. *S. bacillaris* confirmed the significant higher presence in MO samples in comparison with the other treatments, as observed at the harvest time. In the same way, *L. thermotolerans* was also found only in MC samples (9.96%). *M. pulcherrima* was only detected in MC samples (1.26%) while *P. terricola* became appreciable in MNT samples (13.85%), undetected at the harvest time. Other fermentative species, unrevealed at the harvest time, became detectable at this stage of fermentation. In particular, *Candida californica* was found in MO, MC and MNT samples without differences. In addition, *Pichia kluyveri* became detectable in the MNT samples (1.08%).

#### 3.2.2. Culture-Dependent Analysis

**Figure 5:**
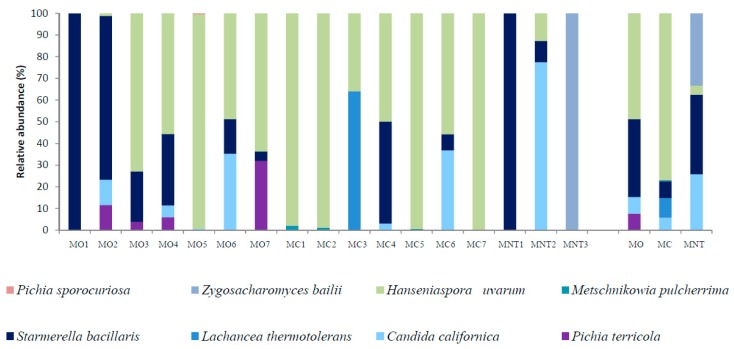
Relative abundance of yeast species detected by the culture-dependent method at the 7th day of spontaneous fermentation in organic (MO), conventional (MC) and not treated (MNT) samples. The number associated to the samples represent the replicates for each treatment. To the right of the graph, mean values of each treatment are represented (MO, MC and MNT).

The results obtained by culture-dependent method after the 7th day are shown in Figure 5. As observed through NGS, *H. uvarum* was the most abundant species in MO and MC samples (48.69% and 76.86% respectively) while its presence was lower in MNT samples (4.21%) where the species mainly detected were *S. bacillaris*, *C. californica* and *Zygosaccharomyces bailii* (undetected with NGS) (36.64%, 25.81% and 33.33%, respectively). *S. bacillaris* and *C. californica* were also found in MO and MC samples with a relative abundance comparable to the NGS results. The results of NGS were also confirmed for *L. thermotolerans* and *M. pulcherrima* in MC samples by the culture-dependent method. Differently, using the culture-dependent method *P. terricola* was only detected in MO samples (7.63%).

### 3.3. Effects of Fungicide Treatments on Fungal Community at 15th Day of Spontaneous Fermentation

#### 3.3.1. Culture-Independent Analysis (NGS)

**Figure 6:**
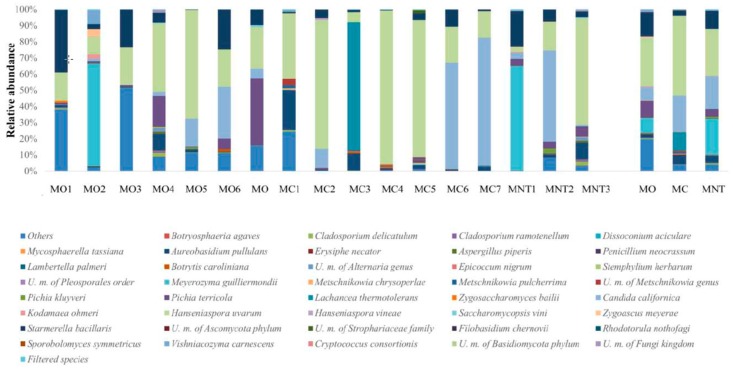
Relative abundance of fungal community detected by NGS at the 15th day of spontaneous fermentation in organic (MO), conventional (MC) and not treated (MNT) samples. The number associated to the samples represents the replicates for each treatment. In the microorganism legend, *U.m.* abbreviation means undefined microorganism. Only the taxa >0.5% are shown and the taxa ≤0.5% were grouped under the heading Filtered species of the legend. To the right of the graph, mean values of each treatment are represented (MO, MC and MNT).

NGS results of the fungal community composition at the 15th day of spontaneous fermentation are shown in Figure 6. The fungal community found was very similar to that found at the 7th day of fermentation. Indeed, at this stage of fermentation 74 species were identified, and *H. uvarum* was confirmed to be the most abundant species detected in all samples, from 29% to 50% of relative abundance. At lower relative abundance, *S. bacillaris* (significant higher in MO samples; Table S1) and *C. californica* were present in all samples. *L. thermotolerans* and *M. pulcherrima* confirmed their presence only in MC samples, *P. kluyveri* was confirmed to be present in MNT samples and *P. terricola* was found in MO and MNT samples (10.17% and 5.01%, respectively). An emerging fermenting species, not detected before, was *Meyerozyma guillermondii* that characterized MO and MNT samples (8.99% and 21.47%, respectively) but was absent in MC samples. High-fermentative yeasts were poorly found (≤0.5%) at this stage of fermentation. In particular, *Torulaspora delbrueckii* in MO and MNT samples and *Z. bailii* in MO and MC samples. *Z. meyerae* remained detectable only in MO samples (0.72%).

#### 3.3.2. Culture-Dependent Analysis

**Figure 7:**
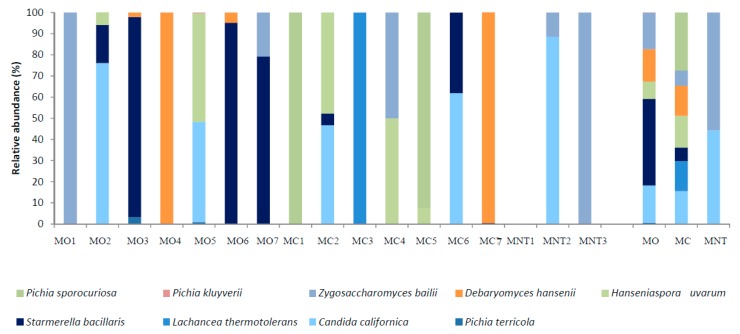
Relative abundance of yeast species detected by culture-dependent method at the 15th day of spontaneous fermentation in organic (MO), conventional (MC) and not treated (MNT) samples. The number associated to the samples represent the replicates for each treatment. To the right of the graph, mean values of each treatment are represented (MO, MC and MNT).

The relative abundances of yeasts by culture-dependent method are shown in Figure 7. The comparison between NGS and conventional methods showed, at this stage of fermentation, some differences in relative abundance since some low fermenting yeasts could be dead or present at a viable but not cultivable condition. Indeed, different to the NGS results, using the culture-dependent method, *H. uvarum* was found at lower relative abundance in all samples (8.18%, 15.06% and not detected in MO, MC and MNT samples respectively). The MNT samples were dominated by two highly fermenting species: *Z. bailii* (55.71%) (just detected by NGS; 0.02%) and *C. californica* (44.30%). The relative abundance of *C. californica* in MO and MC samples was comparable to NGS results. MO samples were dominated by *S. bacillaris* (41.00%) and *Debaryomyces hansenii*, (15.27%) (not detected by NGS). In MC samples, the presence of *L. thermotolerans* was confirmed using both culture-independent and -dependent methods, while the presence of the fermentative yeast *D. hansenii*, not detected by NGS, arose.

### 3.4. Principal Component Analysis

**Figure 8:**
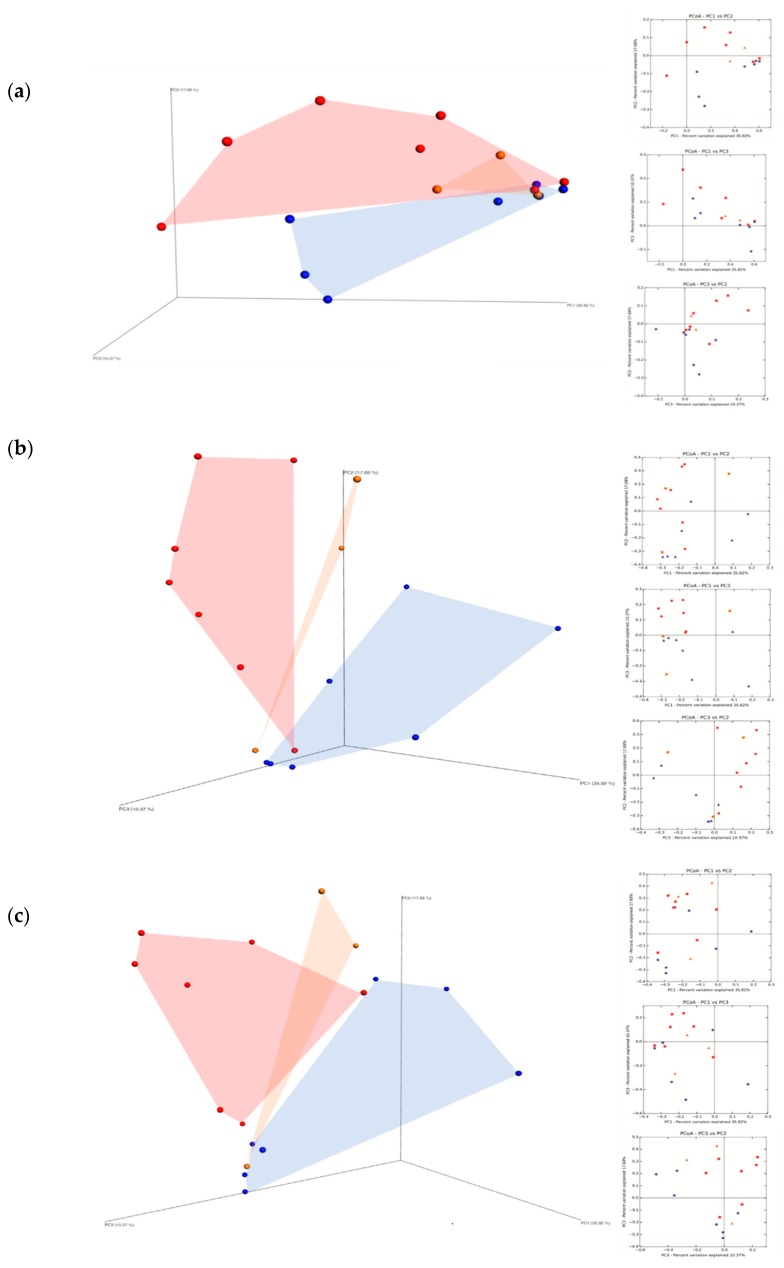
Principal Coordinate Analysis (PCoA) based on the fungal ITS sequences identified in organic (MO) 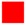; conventional (MC) 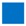 and not treated (MNT) 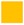 samples at the harvest time (**a**), at 7th day (**b**) and at 15th day (**c**) of spontaneous fermentation. Percentages shown along the axes represent the proportion of dissimilarities captured by the axes.

The fungal community was also subjected to Principal Component Analysis (PCoA) and the distribution of the samples in the three-dimensional plot graphic, at harvest time, at the 7th and 15th day of fermentation, is shown in Figure 8. At harvest time (Figure 8a) the total variance explained was 63.87% (PC 1 35.82%, PC 2 17.68%, PC 3 10.37%). The graphic distribution of the samples highlighted a clear distinction between MO and MC samples, while the MNT samples were closely related to the MO ones.

At the 7th day of spontaneous fermentation, Figure 8b shows a clear distinction in fungal community composition among the three treatments. At the 15th day of spontaneous fermentation MC samples grouped separately, while MO and MNT samples showed some overlaps (Figure 8c).

## 4. Discussion

Knowledge of the complex dynamic microbial ecosystem associated with grape berry surfaces, represented by yeasts, filamentous fungi and bacteria [1], is crucial to better understand their involvement during the winemaking process, with consequent repercussions on wine quality [7,41,42,43]. The study of fungal diversity during the winemaking process using culture-dependent methods can lead to risks such as an incomplete microbial detection and identification, linked to different microbial kinetics or VBNC state of such species or for low abundant strains [44]. Recently, the development of NGS technology allowed to obtain more exhaustive information about microbial communities associated with grape berries, fresh must and wineries [7,29,45,46].

In the present study, the impact of organic and conventional treatments on the occurrence of the fungal community in the Montepulciano variety was evaluated by NGS technology and culture-dependent methods, comparing the overall results obtained. As expected, at the harvest time, 164 fungal species were identified by NGS, while only 14 yeast species were identified by culture-dependent methods. However, both methodologies detected *A. pullulans* and *H. uvarum* as the most abundant species found in all samples at the harvest time. The occurrence of *A. pullulans* seems to be influenced by treatments: indeed, it was the most abundant species detected in conventional samples. These data confirmed the results of previous studies [5,47,48] that found *A. pullulans* as the most abundant species on the grape surface at harvest time. Although this yeast is considered irrelevant in the fermentation process for its inability to ferment sugars, it represents a common resident of grape berries [1]. Regarding *H. uvarum,* no relevant differences at harvest time among the treatments was found using both methodologies.

The presence of *S. bacillaris* characterized MO samples, while *L. thermotolerans* was only found in MC samples. These data are in agreement with those of Ghosh et al. [49] that described *S. bacillaris* as the dominant yeast species in biodynamic Cabernet Sauvignon fresh must and Cordero-Bueso and co-workers [20] that described *L. thermotolerans* as predominant non-*Saccharomyces* yeast found in organic and conventional samples. *M. pulcherrima* was only found in MC and MNT samples using both technologies, confirming the results obtained by Milanović et al. [22] that showed the negative effect of the organic treatments on this species. The fungal dynamic at the 7th day of spontaneous fermentation showed, as expected, a reduction of species and the predominance of *H. uvarum* using both methodologies [4,29,50,51]. However, an overestimation of this yeast using NGS methodology at the 15th day of fermentation was found since the culture-dependent method detected absence or limited presence in all samples. This result could be due to the detection by NGS method of dead and/or viable but non culturable cells showing, at this time, a warped picture of the fermentative yeast population. Indeed, at this time, using the culture-dependent method, a predominance of *S. bacillaris*, *C. californica* and *Z. bailii* in MNT samples was found. The fermenting yeasts *C. californica* and *P. kluyveri*, seemed to be negatively influenced by treatments. Other fermenting yeasts (*M. guillermondii*, *T. delbrueckii* and *Z. meyerae*) were detected in MO and/or in MNT samples only by NGS analysis. *D. hansenii* (MO and MC samples) and *Z. bailii* (MNT samples) were detected only by the culture-dependent method. It is necessary underline that the failure in some species identification by NGS, could be due to a significant portion of relative abundance described as unidentified yeasts or fungi. In this regard, the choice of the target used during microbial metabarcoding and the availability of an exhaustive reference database for the target chosen becomes very important. To date, only few fungal databases are available, therefore more exhaustive database information of bioinformatics packages could be necessary to improve the sensitivity of the method [52,53]. In the condition tested, *S. cerevisiae* was very poorly detected and only using the NGS method (about 0.003% at the 7th and 15th day of spontaneous fermentation) in agreement with previous works [54,55,56]. Similar to previous investigations [22,23,24,25,26,27,28,29,30,31,32,33,34,35,36,37,38,39,40,41,42,43,44,45,46,47,48,49,50,51,52,53,54,55]. , carried out in the same conditions (aseptically microfermentations), relevant residual sugars and high volatile acidity were found, probably influencing the poor finding of *S. cerevisiae* (Tables S3 and S4).

The two methods used in the present study revealed the same species concerning the dominant yeast species even if with different relative abundances (overestimation of *H. uvarum* at the 7th and 15th day of fermentation). This result highlights some limitations of NGS methodology regarding its application in the monitoring of the yeast dynamic during the fermentation process. On the other hand, the NGS method was able to identify a great biodiversity in comparison with the culture-dependent method, particularly regarding the lower abundant species.

The influence of fungicide treatments on the grape yeast community composition highlighted the loss of yeast biodiversity with conventional treatments in agreement with the results of Cordero-Bueso et al. [20] and Escribano-Viana et al. [23]. Chemical compounds (conventional treatments) seem to adversely affect the fermenting yeasts in favor of oxidative yeasts such as *A. pullulans*. Moreover, *S. bacillaris* was more present in MO samples (detected by NGS method) while *H. uvarum* was significantly lower in MNT samples (culture-dependent method). In this regard, the fermenting yeast species, often undetected at harvest time, become detectable during the fermentation process and are, as a result, different as a function of the fungicide treatments (MC, MO or MNT) applied. Considering the evidence that the yeast community of grape berries plays an important role in the winemaking process, also determining an imprint in relation to geographic viticultural area, the vineyard farming system affecting the mycobiota plays an indirect impact on wine fermentation.

## Supplementary Materials

The following are available online at https://www.mdpi.com/2076-2607/7/5/114/s1, Table S1: Analysis of variance (ANOVA) of filamentous fungi detected by NGS in Montepulciano samples at harvest time and at the 7th and 15th day of microfermentation carried out using aseptically pressed grapes. Table S2: Analysis of variance (ANOVA) of yeast species detected by culture-dependent method in Montepulciano samples at harvest time and at the 7th and 15th day of microfermentation carried out using aseptically pressed grapes. Table S3: The main analytical compounds of Montepulciano microfermentations carried out using aseptically pressed grapes. Table S4: Analytical results of organic (MO), conventional (MC) and not treated (MNT) Montepulciano samples.
