# Supplementary material for: The Influence of Fungicide Treatments on Mycobiota of Grapes and Its Evolution During Fermentation Evaluated by Metagenomic and Culture-Dependent Methods
Source: Microorganisms. 2019 Apr 26;7(5):114. doi: 10.3390/microorganisms7050114 (PMC6560393; doi:10.3390/microorganisms7050114)
Supplement: Supplementary file 1 [file microorganisms-07-00114-s001.pdf]

microorganisms MDPI

Table S1. Analysis of variance (ANOVA) of filamentous fungi detected by NGS in Montepulciano samples at harvest time and at 7th and 15th day) of microfermentations carried out using aseptically pressed grapes.

Culture-Independent Method (NGS)	Harvest Time			7th Day			15th Day		
**Fungal Species**	**MO**	**MC**	**MNT**	**MO**	**MC**	**MNT**	**MO**	**MC**	**MNT**
Aspergillus piperis	*	nd	nd	A	nd	A	A	nd	A
Aureobasidium pullulans	B	A	A	A	A	A	A	A	A
Botryosphaeria agaves	A	A	A	A	B	AB	A	B	AB
Botrytis caroliniana	A	A	A	A	A	A	A	A	A
Candida californica	nd	nd	nd	A	A	A	A	A	A
Cladosporium delicatulum	A	A	A	A	A	A	A	A	A
Cladosporium ramotenellum	A	B	B	A	B	AB	A	A	A
Cryptococcus consortionis	B	A	AB	nd	nd	nd	nd	nd	nd
Dissoconium aciculare	nd	nd	*	nd	nd	nd	nd	nd	nd
Epicoccum nigrum	A	A	A	A	A	A	A	A	A
Erysiphe necator	B	AB	A	nd	nd	nd	nd	nd	nd
Filobasidium chernovii	A	A	A	A	A	A	A	A	A
Hanseniaspora uvarum	A	A	A	A	A	A	A	A	A
Hanseniaspora vineae	nd	nd	nd	nd	nd	nd	nd	*	nd
Kodamaea ohmeri	nd	nd	nd	nd	nd	nd	A	nd	A
Lachancea thermotolerans	nd	*	nd	nd	*	nd	nd	*	nd
Lambertella palmeri	nd	nd	*	nd	nd	nd	nd	nd	nd
Metschnikowia chrysoperlae	nd	nd	nd	nd	*	nd	nd	*	nd
Metschnikowia pulcherrima	nd	nd	*	nd	*	nd	nd	*	nd
Meyerozyma guilliermondii	nd	nd	nd	nd	nd	nd	A	nd	A
Mycosphaerella tassiana	A	A	A	A	A	A	A	A	A
Penicillium neocrassum	nd	nd	*	nd	nd	nd	nd	nd	nd
Pichia kluyveri	nd	nd	nd	nd	nd	*	nd	nd	*
Pichia terricola	A	A	nd	A	A	A	A	nd	A
Rhodotorula nothofagi	nd	nd	*	nd	nd	nd	nd	*	nd
Saccharomycopsis vini	*	nd	nd	*	nd	nd	A	nd	A
Sporobolomyces symmetricus	B	B	A	A	nd	A	A	nd	A
Starmerella bacillaris	A	B	AB	A	B	AB	A	B	AB
Stemphylium herbarum	A	A	A	A	A	A	A	A	A

U. m. of Alternaria genus	A	A	A	A	A	A	A	A	A
U. m. of Ascomycota phylum	A	A	A	nd	nd	nd	nd	nd	nd
U. m. of Basidiomycota phylum	C	B	A	nd	nd	nd	A	A	A
U. m. of Fungi kingdom	A	A	A	A	A	A	A	A	A
U. m. of Metschnikowia genus	nd	*	nd	nd	*	nd	nd	*	nd
U. m. of Pleosporales order	B	B	A	nd	nd	nd	nd	nd	nd
U. m. of Strophariaceae family	nd	nd	nd	nd	nd	nd	nd	*	nd
Vishniacozyma carnescens	A	A	A	A	A	A	A	A	A
Zygoascus meyerae	A	nd	A	A	nd	A	*	nd	nd
Zygosaccharomyces bailii	A	nd	A	*	nd	nd	A	A	nd

The significant differences were determined using t-Test (*P* value < 0.05). Different letters (A, B, C) within each row indicate significant difference. Only the fungal species > 0.5% of relative abundance were represented. nd = not detected. * = fungi detected in only one treatment (MO, MT or MNT).

Table S2. Analysis of variance (ANOVA) of yeast species detected by culture-dependent method in Montepulciano samplesat harvest timeand at 7th and 15th day of microfermentations carried out using aseptically pressed grapes.

**Culture-Dependent Method**	**Harvest Time**			**7th Day**			**15th Day**		
**Yeast Species**	**MO**	**MC**	**MNT**	**MO**	**MC**	**MNT**	**MO**	**MC**	**MNT**
Aureobasidium pullulans	A	A	A	nd	nd	nd	nd	nd	nd
Candida californica	*	nd	nd	A	A	A	A	A	A
Cryptococcus carnescens	*	nd	nd	nd	nd	nd	nd	nd	nd
Cryptococcus flavescens	nd	nd	nd	nd	nd	nd	nd	nd	nd
Cryptococcus genus	A	A	nd	nd	nd	nd	nd	nd	nd
Debaryomyces hansenii	nd	*	nd	nd	nd	nd	A	A	nd
Hanseniaspora uvarum	A	A	A	AB	A	B	A	A	nd
Lachancea thermotolerans	nd	*	nd	nd	*	nd	nd	*	nd
Metschnikowia pulcherrima	nd	B	A	nd	*	nd	nd	nd	nd
Pichia kluyveri	nd	nd	nd	nd	nd	nd	*	nd	nd
Pichia sporocuriosa	nd	nd	nd	*	nd	nd	nd	*	nd
Pichia terricola	A	A	A	*	nd	nd	*	nd	nd
Rhodothorula genus	nd	A	nd	nd	nd	nd	nd	nd	nd
Starmerella bacillaris	A	nd	A	A	A	A	A	A	A
Zygoascus meyerae	A	nd	A	nd	nd	nd	nd	nd	nd
Zygosaccharomyces bailii	nd	nd	*	nd	nd	*	A	A	A

The significant differences were determined using t-Test (P value <0.05). Different letters (A, B) within each row indicate significant difference. Only the yeast species > 0.5% of relative abundance were represented. nd = not detected. * = fungi detected in only one treatment (MO, MT or MNT).

microorganisms MDPI

Table S3. The main analytical compounds of Montepulciano microfermentations carried out using aseptically pressed grapes.

Samples	Residual Sugars * (g L^{-1})	Ethanol (% *v*/*v*)	Volatile Acidity (as Acetic Acid g L^{-1})
MO1	44.7	9.9	6.00
MO2	66.2	8.7	2.76
MO3	58.1	9.1	4.80
MO4	74.7	8.2	13.92
MO5	81.9	7.8	1.75
MO6	79.1	7.9	5.76
MO7	42.3	10.1	16.32
MC1	43.9	9.9	6.72
MC2	22.5	11.1	1.03
MC3	22.4	11.1	1.73
MC4	65.9	8.7	5.04
MC5	58.4	9.1	4.80
MC6	94.6	7.1	3.36
MC7	92.6	7.2	3.36
MNT1	36.4	10.3	14.40
MNT2	4.3	12.1	2.06
MNT3	24.3	11.0	6.00

* Total initial sugars 221 g $L^{-1.}$

Table S4. Analytical results of organic (MO), conventional (MC) and not treated (MNT) Montepulciano samples.

Samples	Residual Sugars* (g L^{-1})	Ethanol (% *v*/*v*)	Volatile Acidity (as Acetic Acid g L^{-1})
MO	63.86 ± 16.06^{a}	$8.80 \pm 0,90^{b}$	7.33 ± 5.581^{a}
MC	57.20 ± 29.78^{a}	9.17 ± 1.67^{b}	3.72 ± 1.97^{a}
MNT	21.68 ± 16.23^{b}	11.16 ± 0.91^{a}	7.49 ± 6.30^{a}

* Total initial sugars 221 g L^{-1}. Data are means ± standard deviations. Data with different superscript letters (^{a,b}) within each column are significantly different (Duncan test; $p < 0.05$).

© 2019 by the authors. Submitted for possible open access publication under the terms and conditions of the Creative Commons Attribution (CC BY) license (http://creativecommons.org/licenses/by/4.0/).
